# Editorial: Sexuality and sexual dysfunctions

**DOI:** 10.3389/fpsyg.2026.1807428

**Published:** 2026-02-26

**Authors:** F. Javier Del Río Olvera, María Del Mar Sánchez-Fuentes, Samantha Banbury

**Affiliations:** 1 Department of Psychology, University of Cádiz, Cádiz, Spain; 2 Department of Personality, Assessment, and Psychological Treatment, School of Psychology, Universidad de Granada, Granada, Spain; 3 London Metropolitan University, London, United Kingdom

**Keywords:** editorial, men sexual health, sexual dysfunction, sexual health, sexuality, women sexual health

In an era in which access to information is greater than ever, the general population paradoxically holds increasing levels of misinformation about specific topics. This is particularly evident in the field of sexuality. Today, individuals often seek information on the internet that may originate from sources of questionable reliability, resulting in content that can be confusing or inaccurate. For this reason, scientifically grounded, evidence-based information becomes especially relevant. The aim of this Research Topic has been to provide accurate and useful information on this topic, both for the general population and for professionals, by presenting a diverse and compelling collection of articles that will equip professionals with practical tools and foster interest in sexological science among the public.

An essential aspect of science is the availability of reliable and valid measurement tools that allow for accurate assessment of sexological variables in individuals, and this Research Topic includes three articles on this topic. The article *Psychometric evaluation of the abbreviated Hungarian Faking Orgasm Scale for Women* (Csányi et al.) examines the factorial structure of a scale designed to explore the underlying motivation in cases of faked orgasm. The article *Development of a cross-cultural scale on attitudes toward gender and sexual diversity* (AGSD) (Oleas et al.) presents the development of a scale aimed at assessing attitudes toward gender and sexual diversity. This scale constitutes a valuable tool for both clinical and educational contexts, where attitudes toward gender and sexual diversity can be evaluated to promote changes toward healthier attitudes. The third article focused on instrument validation is entitled *Validation of the Brief Index of Sexual Functioning for women and men (BISF-W and BISF-M) in an Italian sample* (Panzeri et al.). This study validates an assessment instrument originally designed for women and develops a version for men, in its Italian adaptation, to evaluate sexual experiences in both clinical and experimental settings.

Additionally, this Research Topic features several articles with a clear clinical orientation, such as *Influence of personality disorders on sexual behaviors and response to treatment of psychogenic erectile dysfunction in phosphodiesterase 5 inhibitor non-responders* (Cabello-García et al.), which analyses the influence of personality disorders on erectile dysfunction. This Research Topic remains under-researched and is of considerable clinical interest in the treatment of patients who do not respond to pharmacological interventions.

The Research Topic also includes the article *Psychological and sociodemographic factors associated with hypoactive sexual desire in Ecuadorian women* (Pérez-Vega et al.), which examines hypoactive sexual desire in Ecuadorian women according to Kaplan's model. This article highlights the importance of maintaining a positive sexual attitude in fostering higher levels of sexual desire.

Another important contribution of this Research Topic lies in its focus on help-seeking for sexual difficulties and the barriers that may hinder access to professional support. Specifically, it includes the articles *Formal help-seeking among community-based czech individuals with sexual interest in minors is associated with the perceived urgency of self-identified concerns* (Martinec Nováková et al.), *Silent struggles: help-seeking barriers for sexual difficulties among adults aged 50 and older in Czechia* (Gore-Gorszewska et al.), and *Sexual distress with partnered face-to-face sexual activity: an exploratory qualitative study with heterosexual cisgender individuals who seek and do not seek professional help* (Pascoal et al.).

Finally, the Research Topic is further enriched by a set of articles addressing diverse and emerging topics within the field of sexuality. The article *Educational intervention on sexual satisfaction of iranian men: application of the information, motivation, and behavioral skills model* (Ghaderi et al.) provides evidence on an educational intervention delivered through an online platform. The article *Masturbation parameters: their relation to sexual arousal in young people who engage in same-sex relationships* (Sánchez-Pérez et al.) highlights gender differences in masturbation among young individuals engaged in same-sex relationships. The final article, *Sexual desire for non-normative sexual behaviors: differences between centennials and millennials considering sexual orientation* (Paramio et al.), addresses concerns related to sexual behaviors in young people within non-psychopathological contexts.

Overall, this Research Topic constitutes a highly informative and timely contribution for researchers and clinicians seeking to stay up to date in the field of sexuality and sexual dysfunctions.

